# AZD6738 (Ceralasertib) Enhances Trifluridine's Antitumor Effect in 5‐FU–Resistant Colorectal Cancer Cells

**DOI:** 10.1002/cnr2.70668

**Published:** 2026-09-01

**Authors:** Shuhei Uehara, Takuya Suzuki, Junki Kato, Hiroyuki Asai, Akira Kato, Shinnosuke Harata, Hajime Ushigome, Yushi Yamakawa, Takahisa Hirokawa, Hiroki Takahashi, Yoichi Matsuo, Shuji Takiguchi

**Affiliations:** ^1^ Department of Gastroenterological Surgery Nagoya City University Graduate School of Medical Sciences Nagoya Japan; ^2^ Department of Gastroenterological Surgery, East Medical Center Nagoya City University Graduate School of Medical Sciences Nagoya Japan; ^3^ Department of Gastroenterological Surgery Kariya Toyota General Hospital Kariya Japan; ^4^ Department of Gastroenterological Surgery, West Medical Center Nagoya City University Graduate School of Medical Sciences Nagoya Japan

**Keywords:** 5‐FU resistance, ATR inhibitor, colorectal cancer, trifluridine (FTD)

## Abstract

**Background:**

Resistance to 5‐fluorouracil (5‐FU) remains a major obstacle in colorectal cancer (CRC) treatment. Trifluridine (FTD), the active component of TAS‐102, exerts cytotoxicity through DNA incorporation and reportedly remains active in 5‐FU‐resistant tumors, though with limited clinical efficacy.

**Aims:**

We investigated whether AZD6738, an inhibitor of ataxia telangiectasia and Rad3‐related kinase (ATR) that regulates the G2/M checkpoint, could enhance FTD efficacy in 5‐FU‐resistant CRC.

**Methods and Results:**

Parental and 5‐FU‐resistant HCT116 (p53 wild‐type) and DLD‐1 (p53 mutant‐type) sublines were evaluated using WST‐1 viability assays, flow cytometry, and immunoblotting for γH2AX, cleaved caspase‐3, and p‐Chk1 (Ser345). Antitumor efficacy and safety of TAS‐102 + AZD6738 were examined in a DLD‐1/5FUR xenograft model by measuring tumor volumes and weights, body and organ weights, blood counts, and γH2AX immunohistochemistry. A noncytotoxic AZD6738 concentration significantly enhanced FTD cytotoxicity across a wide concentration range in both parental and resistant cells. The combination increased γH2AX and cleaved caspase‐3 while suppressing FTD‐induced Chk1 phosphorylation, findings consistent with increased DNA damage and apoptosis following pharmacological treatment with AZD6738. Flow cytometry analysis revealed that AZD6738 abrogated FTD‐induced G2/M arrest. In vivo, TAS‐102 + AZD6738 significantly suppressed tumor growth compared with monotherapy without increasing hematologic or systemic toxicity.

**Conclusion:**

AZD6738 enhances the antitumor activity of FTD in preclinical models of 5‐FU‐resistant CRC and is associated with increased DNA damage and apoptosis. These findings provide proof‐of‐concept supporting further mechanistic and translational investigation of this combination.

**Abbreviations**
ATR: ataxia telangiectasia and Rad3‐related kinase
AZD6738: Ceralasertib
CRC: colorectal cancer
FTD: trifluridine
FUR: fluorouracil resistant
TAS‐102: trifluridine + tipiracil
TS: thymidylate synthase

## 1 Introduction

Colorectal cancer (CRC) is one of the leading causes of cancer‐related death worldwide, having high incidence and mortality. According to the International Agency for Research on Cancer 2020 report, CRC accounted for the third‐highest number of new cases (approximately 1.93 million) among all cancers, with about 0.94 million deaths attributed to it [1]. In particular, in developed countries, its incidence has been increasing together with changes in lifestyle and diet [2]. In Japan, the number of deaths from CRC increases every year, ranking second among men and first among women [3].

The treatment for CRC involves combinations of surgery, chemotherapy, radiotherapy, molecularly targeted therapy, and immunotherapy according to disease stage [4]. For locally advanced disease, surgery plus adjuvant chemotherapy is the standard of care, whereas systemic chemotherapy is the mainstay for recurrent or metastatic disease. Currently, treatments based on 5‐fluorouracil (5‐FU) such as FOLFOX and FOLFIRI, combined with molecularly targeted agents including the anti‐VEGF antibody bevacizumab or the anti‐EGFR antibodies cetuximab and panitumumab, have improved overall survival [5, 6, 7, 8]. Nevertheless, challenges remain, including chemoresistance acquisition, serious adverse events, and limited benefits in a subset of patients. Therefore, there is an urgent need for new therapeutic drugs.

Recently, the DNA damage checkpoint—an intrinsic repair network activated in response to DNA‐damaging anticancer agents—has attracted attention. In this network, sensor protein complexes detect DNA lesions and activate downstream signaling to halt the cell cycle, thereby preventing replication and propagation of damaged DNA. Generally, this system comprises the G1 checkpoint controlled by the ATM‐p53‐p21 pathway and the G2/M checkpoint controlled by the ataxia telangiectasia and Rad3‐related kinase (ATR)‐Chk1‐Cdc25 pathway. As malignancies frequently harbor p53 mutations and do not enter G1 arrest, they are considered to rely on the G2/M checkpoint to cope with DNA damage [9, 10]. Accordingly, therapeutically targeting the G2/M checkpoint may have selective anticancer effects, as the ATM‐p53‐p21 pathway remains functional in normal cells [11, 12].

AZD6738 (Ceralasertib) inhibits ATR‐dependent phosphorylation, most notably Chk1 phosphorylation at Ser345, thereby suppressing the G2/M checkpoint [13, 14, 15]. In gastrointestinal cancer, preclinical studies have focused on gastric [14], pancreatic [16], and biliary tract cancer [17]. In addition, a Phase II trial suggested promising clinical activity for a combination of AZD6738 with durvalumab in previously treated advanced gastric cancer, particularly in the patient subgroup lacking ATM expression—a regulator of the G1 checkpoint [18]. In nonsmall cell lung cancer, a Phase II study demonstrated efficacy of a combination with durvalumab, an anti‐PD‐L1 antibody [19]. Further, emerging data suggest effects of combinations with the PARP inhibitor olaparib in triple‐negative breast cancer or durvalumab in melanoma [20, 21]. Recently, combinations with radiotherapy are attracting increasing interest [22].

However, AZD6738 has yet to be implemented in routine clinical practice. So far, there are few preclinical studies applying it to CRC. Previously, based on the potential of AZD6738 for increasing the activity of established agents in CRC, we demonstrated that AZD6738 enhances the antitumoral effects of 5‐FU [23]. Then, we turned our attention to trifluridine (FTD), the principal active ingredient of TAS‐102. FTD is directly incorporated into DNA during S phase instead of thymine and induces DNA damage [24]. Although FTD has been reported to result in higher DNA incorporation than 5‐FU [25], its tumor‐shrinking effect is limited in practice and it is used as third‐line therapy or later for unresectable or recurrent CRC, generally in combination with a molecular‐targeted agent (bevacizumab) [26, 27, 28]. Thus, enhancing the therapeutic efficacy of FTD in later‐line treatment may contribute to improving survival in patients with advanced CRC. Accordingly, we hypothesized that combining AZD6738 with FTD would enhance therapeutic efficacy and reported the potentiating effect of AZD6738 on FTD [29]. Since FTD induces extensive DNA incorporation, which causes replication stress, ATR inhibition represents a rational approach to potentiate its cytotoxicity.

Clinically, the acquisition of 5‐FU resistance explains well the limited benefit of later‐line chemotherapy in CRC [30]. Since FTD is typically administered after first‐ and second‐line 5‐FU‐based regimens, a substantial portion of tumors already harbor 5‐FU resistance. This clinical context raises a key therapeutic question: does ATR inhibition enhance the efficacy of FTD in tumors that have survived prolonged 5‐FU exposure and adapted to genotoxic stress? To address this clinically relevant question, we employed genetically defined 5‐FU–resistant CRC cell lines as a model of the treatment‐refractory setting. Our aim was to clarify the potentiating effect of AZD6738, an ATR inhibitor targeting the DNA damage checkpoint, on FTD in a clinical context characterized by preexisting resistance to 5‐FU.

## 2 Materials and Methods

### 2.1 Cell Lines and Culture Conditions

HCT116, a human CRC cell line (ATCC CCL‐247), was obtained from the American Type Culture Collection. Cells were grown in DMEM (FUJIFILM Wako Pure Chemical Corporation) containing 10% FBS (Gibco, Thermo Fisher Scientific) and 1% penicillin/streptomycin. All cultures were maintained under standard conditions at 37°C in a humidified atmosphere of 5% CO_2_. The 5‐FU–resistant derivative HCT116/5FUR was established following published methods [31] [32] by stepwise escalation of 5‐FU from 0.2 μM for > 100 passages. Compared with the parental line, the selected subclone exhibited approximately 100‐fold higher IC50 for 5‐FU.

Both HCT116 and HCT116/5FUR cells were authenticated by short tandem repeat (STR) profiling performed at the Japanese Collection of Research Bioresources Cell Bank (authentication no. KBN0950).

Both DLD‐1 (ATCC, CCL‐221) and 5‐FU–resistant DLD‐1/5FUR cell lines were provided by Taiho Pharmaceutical, with material transfer permitted by ATCC. Similarly, DLD‐1/5FUR was established by stepwise exposure over > 100 passages and authenticated by STR‐PCR. DLD‐1 cells were maintained in RPMI‐1640 medium containing 10% FBS and 1% penicillin/streptomycin.

To remove the influence of multiple passages, the parental HCT116 and DLD‐1 cells used in this study were subcultured to a similar extent as their corresponding resistant sublines. To exclude acute drug effects, cells were cultured drug‐free for ≥ 3 passages before experiments.

All cell lines were tested for Mycoplasma contamination using a MycoAlert Mycoplasma detection kit (Lonza Group Ltd.) and confirmed as Mycoplasma‐free before all experiments.

We designed four groups for in vitro experiments: Control, AZD6738, 5‐FU, and 5‐FU plus AZD6738, or alternatively, Control, AZD6738, FTD, and FTD plus AZD6738. In animal experiments, TAS‐102 was used as the FTD source.

AZD6738 (Selleck; S7693) was used at 0.5 μM. At this concentration, AZD6738 did not affect proliferation after 72 h in any cell line. 5‐FU (FUJIFILM Wako Pure Chemical Corporation) was used at 4 μM for HCT116 and HCT116/5FUR, and at 7 μM for DLD‐1 and DLD‐1/5FUR; these concentrations correspond to the IC50 values after 72 h treatment in each parental cell line. FTD (Tokyo Chemical Industry; T2511‐1G) was used at 5 μM for HCT116 and HCT116/5FUR, and at 30 μM for DLD‐1 and DLD‐1/5FUR; these concentrations approximate the IC50 values after 72 h treatment in each parental cell line. Dose–response curves and IC50 values are presented in Figure S1. We selected these concentrations to assess whether a noncytotoxic AZD6738 dose could potentiate the effects of the combined agents.

### 2.2 WST‐1 Assays

The Premix WST‐1 Cell Viability Assay System (Takara Bio) was employed to determine cell viability. For viability assays, cells were plated in 96‐well plates at 5.0 × 10^3^ cells per well and cultured overnight before drug administration. Six wells were used for each experimental condition. In experiments testing a gradient of concentrations at the following ranges: 5‐FU (0–1000 μM), FTD (0–1000 μM), and AZD6738 (0–10 μM), absorbance was measured after 72 h. In experiments including the four groups described above, absorbance was measured at 0, 24, 48, and 72 h after drug administration. Following the addition of WST‐1 reagent, plates were incubated for 1 h, after which optical density at 450 nm was determined using a SpectraMax ABC microplate reader (Molecular Devices). The IC50 was calculated by interpolation using the equation 10^[log_10_(*A*/*B*) × (50 − *D*)/(*C* − *D*) + log_10_(*B*)], where *A* and *B* are the two concentrations flanking the 50% inhibition point and *C* and *D* are the corresponding percentages of growth inhibition observed at those concentrations.

### 2.3 Flow Cytometry

HCT116, HCT116/5FUR, DLD‐1, and DLD‐1/5FUR cells were divided into four groups and harvested at 0, 24, 48, and 72 h after each reagent administration followed by fixation in 70% ethanol. For cell‐cycle assessment, samples were processed using the BD Cycletest Plus DNA Kit and examined with a FACSCanto II flow cytometer (BD Biosciences). Flow cytometric data were analyzed using BD FACS Diva software (version 8.0.2).

### 2.4 Western Blotting

Cell lysates were prepared in RIPA buffer supplemented with protease/phosphatase inhibitors. Protein concentrations were quantified using a BCA Protein Assay Kit (Thermo Fisher Scientific). For immunoblotting, 20 μg of protein per sample was heated at 90°C for 5 min and resolved on Mini‐PROTEAN TGX SDS–polyacrylamide gels. Proteins were subsequently transferred onto nitrocellulose membranes (Bio‐Rad). Membranes were processed using the iBind Flex Western System together with the iBind Flex Solution Kit (Thermo Fisher Scientific) and incubated with primary antibodies followed by the appropriate secondary antibodies for 4–6 h at room temperature.

Primary antibodies used were Chk1 (1:1000; C9358, MilliporeSigma), phospho‐Chk1 (Ser345) (1:1000; 2348, Cell Signaling Technology), cleaved caspase‐3 (Apoptosis Western Blot Cocktail; 1:250; ab136812, Abcam), H2AX (1:1000; ab11175, Abcam), and γH2AX (1:2000; 05–636, Merck KGaA), GAPDH (1:1000; 14C10, Cell Signaling Technology). Secondary antibodies used were: for Chk1 and γH2AX, HRP‐conjugated goat anti‐mouse immunoglobulins (1:2000; P0447, Agilent Technologies); for p‐Chk1, H2AX, and GAPDH, HRP‐conjugated goat anti‐rabbit immunoglobulins (1:1000–1:2000; P0448, Agilent Technologies); and for cleaved caspase‐3, Apoptosis Western Blot Cocktail secondary antibodies (1:100; ab136812, Abcam). Immunoreactive bands were detected by enhanced chemiluminescence using SuperSignal West Pico or Femto substrates (Thermo Fisher Scientific). Images were acquired with an Amersham Imager 680 system (GE Healthcare Life Sciences). Bands were semiquantified by densitometric analysis using ImageJ version 1.54 (National Institutes of Health).

### 2.5 Animal Experiments

Twenty‐four male BALB/c nu/nu mice (6 weeks old) were purchased from Japan SLC Inc. Six mice were housed per standard Plexiglas cage under controlled temperature (20°C–26°C) and humidity (40%–60%) with a 12‐h light/dark cycle, with ad libitum access to autoclaved chow and water. Individual identification numbers (Natsume Seisakusho; KN‐295) were attached to the left auricle using the ear‐punch method. After a 2‐week acclimation period, mice were anesthetized with 2% isoflurane (prod. no. 099‐06571; FUJIFILM Wako Pure Chemical) using a Rodent Circuit Controller (vetequip), and DLD‐1/5FUR cells (3 × 10^6^ in 200 μL PBS) were injected subcutaneously into the right flank. Subcutaneous tumor formation was confirmed in all mice. Then, animals were randomized into four groups (*n* = 6 each): control, AZD6738, TAS‐102, and TAS‐102 + AZD6738.

Drug administration began when the mean tumor volume reached 50 mm^3^. Oral dosing was performed using a syringe (Terumo; IC‐1‐4908‐01) and an 18G oral gavage needle (Natsume Seisakusho; vS‐493‐18GS). Control animals received equal volumes of the corresponding vehicles. TAS‐102 combines FTD with tipiracil, an inhibitor of FTD catabolism in the liver, to enable oral administration [33], and is clinically known as Lonsurf. Following its package information, FTD and tipiracil hydrochloride (MCE HY‐A0063) were mixed at a molar ratio of 1:0.5 (weight ratio 1:0.471) and diluted in 0.5% (w/v) hydroxypropylmethyl cellulose (Shin‐Etsu Chemical) at an FTD‐equivalent concentration of 7.5 mg/mL. In addition, AZD6738 was formulated in a vehicle composed of 5% DMSO, 40% propylene glycol, and 55% sterile distilled water, following the manufacturer's protocol. Doses were based on prior studies: TAS‐102 at an FTD‐equivalent 200 mg/kg/day (100 mg/kg twice daily, 6 h apart) [34] and AZD6738 at 25 mg/kg/day (once daily) [35, 36]. Doses were adjusted based on daily body weight. The dosing period was 2 weeks following a 5‐days‐on/2‐days‐off schedule.

Tumor volume was measured daily and calculated as: volume (mm^3^) = length × width × width/2. At the end of the 2‐week treatment period, mice were anesthetized with 2% isoflurane and euthanized by cervical dislocation. Death was verified by the absence of cardiac activity. Humane endpoints included tumor burden exceeding 10% of body weight, tumor diameter greater than 20 mm, tumor ulceration, necrosis, or infection, impaired mobility, substantial reduction in food or water intake, body‐weight loss exceeding 20% (or > 25% within 7 days compared with controls), and evidence of cachexia. No mouse met these criteria during the study. All mice in each group were necropsied, and the weight of the tumor, liver, kidneys, spleen, and testes recorded. Blood was collected from the inferior vena cava into K2‐EDTA tubes (Kyokuto Pharmaceutical; SPM‐K0501EM, Insepack II‐D), and hematologic testing performed by FUJIFILM Wako Pure Chemical. Animal studies were conducted with the approval of the Animal Experiment Committee of Nagoya City University Graduate School of Medical Sciences (approval no. 23‐049; Nagoya, Japan). The animal housing rooms and experimental facilities of the Nagoya City University Experimental Animal Education and Research Center were equipped with P2A‐level containment measures and certified (certification no. FM3).

### 2.6 Immunohistochemistry

Tumor samples were immersed in 4% paraformaldehyde for 6 h at 4°C, embedded in paraffin, and sectioned to a thickness of 3 μm. Paraffin sections were mounted onto 3‐aminopropyltriethoxysilane‐coated slides for subsequent staining. After hematoxylin–eosin (H&E) staining, automated immunohistochemistry was performed on a Bond Rxm system (Leica Biosystems, Newcastle, UK). The BOND Polymer Refine Detection kit (DS9800) was used as a compact polymer detection system. The primary antibody was anti‐gamma H2A.X (phospho S139) [EP854(2)Y] (1:3000; ab81299, Abcam). Deparaffinization was performed at 72°C with Bond Dewax Solution (AR9222), followed by washing with alcohol and Bond Wash Solution (AR9590). Sections were subjected to antigen retrieval with Bond Epitope Retrieval Solution 1 (AR9961) at 100°C for 20 min. Then, the endogenous peroxidase was blocked for 5 min at room temperature before incubation with the primary antibody for 15 min at room temperature. Next, the secondary antibody was incubated with the Polymer reagent for 8 min at room temperature. After 10 min of DAB chromogenic development, nuclei were counterstained with hematoxylin for 5 min at room temperature.

Fluorescence images were acquired using a BZ‐X710 microscope (Keyence, Osaka, Japan). For each tumor (*n* = 6 per group), 10 high‐power fields at ×200 magnification were imaged, and the mean proportion of nuclear γH2A.X‐positive cells quantified using hybrid cell count software (BZ‐X Analyzer ver. 1.4.0.1; Keyence).

### 2.7 Statistical Analysis

All in vitro experiments were performed at least three times independently. For WST‐1 assays, each condition was analyzed in six technical replicates per experiment. In vivo experiments were performed once. Statistical analyses were conducted using EZR software (Easy R) version 1.61 (Saitama Medical Center, Jichi Medical University, Saitama, Japan). Data are presented as mean ± SD. Two‐group comparisons used the unpaired Student's *t*‐test; for ≥ 3 groups, one‐way ANOVA followed by Tukey's test was applied. *p* < 0.05 was considered to indicate statistical significance.

## 3 Results

### 3.1 Establishment of 5‐FU–Resistant Cells

**FIGURE 1:**
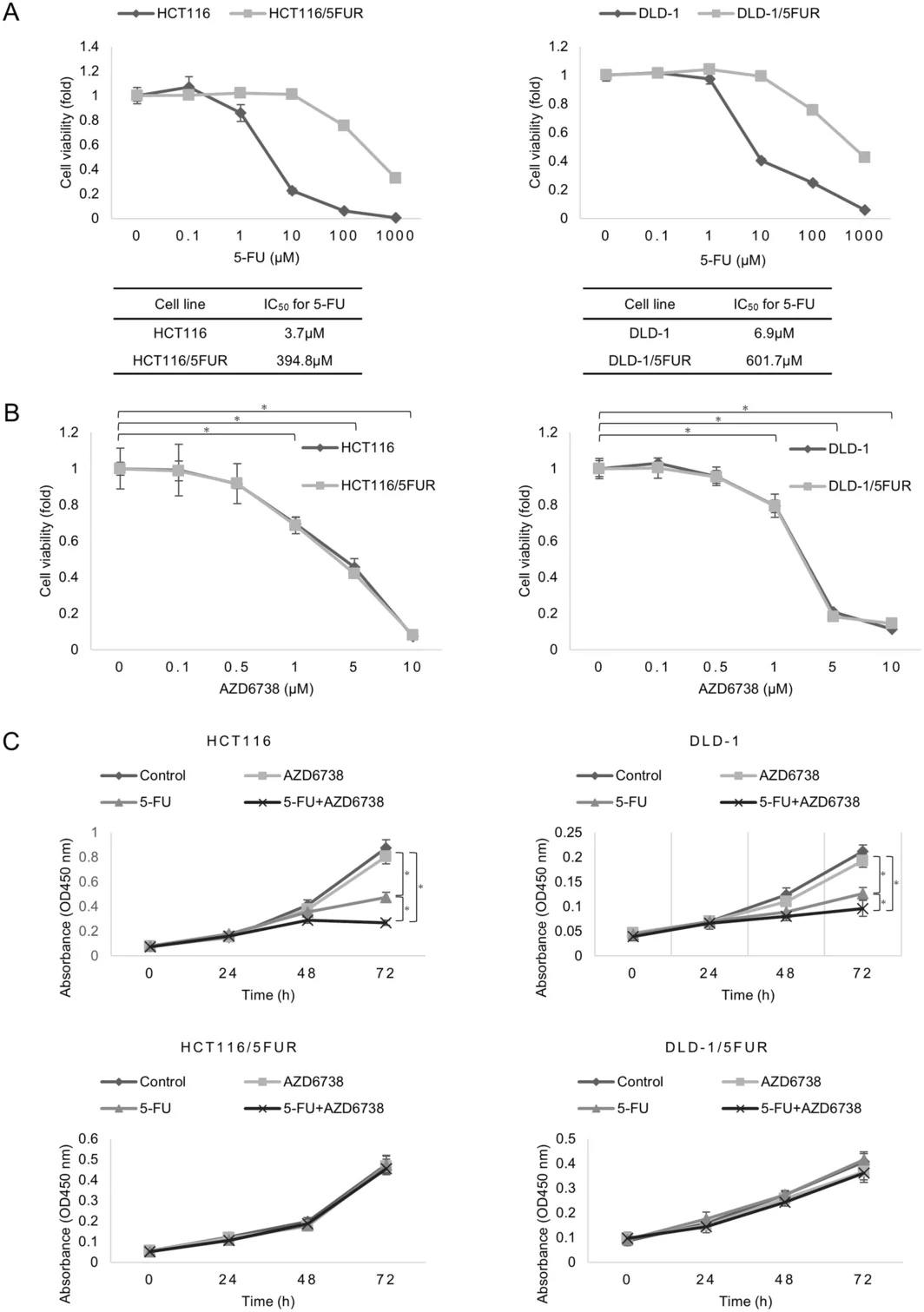
Characterization of 5‐FU–resistant cell lines. (A) WST‐1 assessment of 5‐FU sensitivity in parental versus 5‐FU–resistant CRC cell lines. HCT116, HCT116/5FUR, DLD‐1, and DLD‐1/5FUR cells were treated with 5‐FU for 72 h, and cell viability was quantified by WST‐1. Resistant sublines showed markedly elevated 5‐FU IC50 values—approximately 100‐fold higher—than their respective parental lines. (B) WST‐1 evaluation of AZD6738 cytotoxicity and selection of a working concentration. After 72 h exposure, AZD6738 showed comparable cytotoxicity in parental and 5‐FU–resistant HCT116 and DLD‐1 sublines (HCT116 vs. HCT116/5FUR; DLD‐1 vs. DLD‐1/5FUR). Although concentrations of 1 μM or higher exerted significant cytotoxicity, 0.5 μM AZD6738 did not significantly suppress cell proliferation; accordingly, this noncytotoxic concentration was used in subsequent experiments to assess the potentiating effect of AZD6738 on trifluridine (FTD). Exact *p*‐values for comparison with 0 μM AZD6738 were as follows: 0.5 μM—HCT116, *p* = 0.226; HCT116/5FUR, *p* = 0.668; DLD‐1, *p* = 0.146; DLD‐1/5FUR, *p* = 0.118 (all not significant). 1 μM—HCT116, *p* = 9.0 × 10^−5^; HCT116/5FUR, *p* = 1.2 × 10^−7^; DLD‐1, *p* = 6.8 × 10^−5^; and DLD‐1/5FUR, *p* = 1.5 × 10^−5^ (all significant). Data in (A) and (B) are expressed as values normalized to the corresponding untreated control for each cell line. (C) In a time‐course WST‐1 assay, the combination of AZD6738 at a concentration ineffective as monotherapy with the IC50 concentration of 5‐FU significantly suppressed cell proliferation compared with 5‐FU monotherapy in parental cell lines at 72 h (HCT116, *p* = 3.4 × 10^−6^; DLD‐1, *p* = 0.005). In contrast, neither monotherapy nor combination treatment was effective in resistant cell lines. Data indicate mean ± SD. Asterisks indicate *p* < 0.05. IC50, half‐maximal inhibitory concentration; FUR, fluorouracil resistant.

The degree of resistance in the 5‐FU–resistant sublines was evaluated using the WST‐1 assay. Both HCT116/5FUR and DLD‐1/5FUR exhibited ~100‐fold higher IC50 values than their respective parental cell lines (Figure 1A).

### 3.2 AZD6738 Cytotoxicity

After AZD6738 monotherapy (0–10 μM, 72 h), there was no difference in cell viability between parental and resistant lines. 0.5 μM AZD6738 had no significant effect in either HCT116 or DLD‐1, whereas concentrations ≥ 1 μM did. Therefore, we used 0.5 μM AZD6738 in the subsequent experiments as a concentration that exhibited no cytotoxicity on its own to assess the potentiating effect of AZD6738 in combination with trifluridine (FTD) (Figure 1B).

### 3.3 Combination of 5‐FU and AZD6738

Consistent with Suzuki's results in HCT116, DLD‐1, HT29, and SW480 cells [23], in the parental lines, the combination of 5‐FU at its IC50 concentration with AZD6738 at an ineffective monotherapy dose significantly suppressed cell proliferation at 72 h compared with 5‐FU alone. In contrast, in resistant sublines, neither 5‐FU at this concentration nor its combination with AZD6738 exerted any effect (Figure 1C).

### 3.4 Synergistic Effect of FTD and AZD6738

**FIGURE 2:**
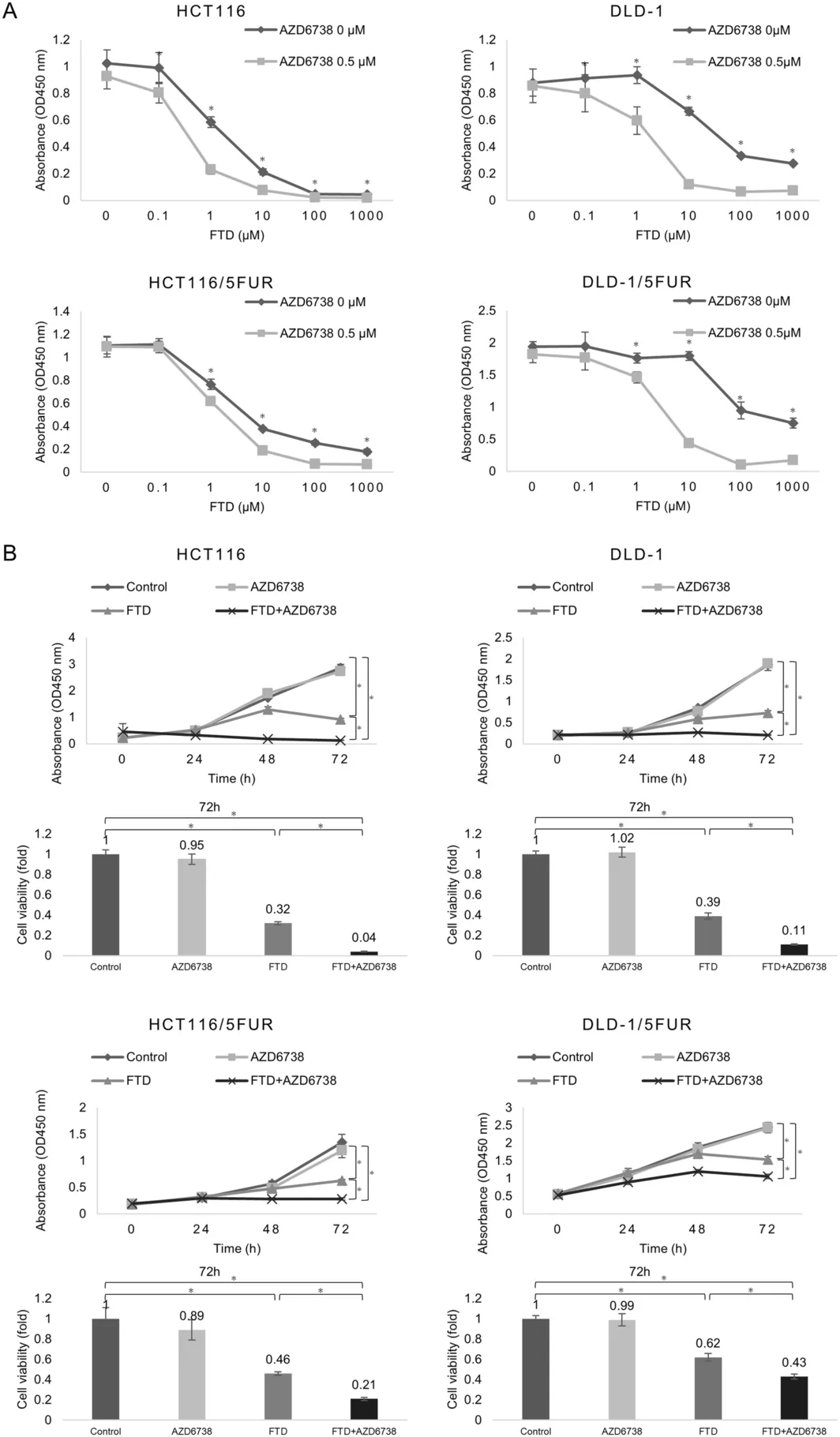
FTD potentiation by AZD6738 in parental and 5‐FU–resistant colorectal cancer cells. (A) HCT116, HCT116/5FUR, DLD‐1, and DLD‐1/5FUR cells were treated for 72 h with the indicated FTD concentrations with or without AZD6738 (0.5 μM). Cell viability was quantified by WST‐1. In both lineages, addition of AZD6738—at a concentration ineffective as a single agent—significantly enhanced the growth‐inhibitory effect of FTD in both parental and resistant cells. FTD IC50 (−AZD6738/+AZD6738): HCT116, 1.6/0.4 μM; HCT116/5FUR, 3.6/1.5 μM; DLD‐1, 41.4/2.5 μM; DLD‐1/5FUR, 155/3.5 μM. Data indicate mean ± SD. Asterisks indicate *p* < 0.05 for AZD6738 0 μM vs. AZD6738 0.5 μM at the same FTD concentration. (B) In a time‐course WST‐1 assay, the combination group showed significantly greater growth inhibition than FTD alone in parental cell lines at 72 h (HCT116, *p* < 1 × 10^−7^; DLD‐1, *p* < 1 × 10^−7^). Similarly, in resistant cell lines, the FTD and AZD6738 combination resulted in significant suppression of cell proliferation compared with FTD alone (HCT116/5FUR, *p* = 6.0 × 10^−5^; DLD‐1/5FUR, *p* = 4.0 × 10^−7^). The bar graph shows cell viability at 72 h normalized to the control (set as 1). In the resistant sublines, FTD monotherapy appeared less effective than in the corresponding parental cell lines. However, the addition of AZD6738 produced a level of growth inhibition that appeared comparable to that observed with FTD monotherapy in the parental cell lines. Data indicate mean ± SD. Asterisks indicate *p* < 0.05. FTD, trifluridine; FUR, fluorouracil resistant.

In the parental lines, as shown by Harata [29] in HCT116, DLD‐1, HT29, and SW480, combining AZD6738 (0.5 μM) with FTD significantly decreased cell viability compared with FTD alone, across a concentration range of 0.1–1000 μM. AZD6738 combined with FTD (1–1000 μM) significantly reduced cell viability in HCT116/5FUR and DLD‐1/5FUR. Combination index [37] analysis demonstrated synergistic interactions in both HCT116/5FUR (0.735) and DLD‐1/5FUR (0.418). In the time‐course WST‐1 assay, adding AZD6738 at a noncytotoxic concentration (0.5 μM) enhanced the effect of FTD in both parental and 5‐FU–resistant cell lines, resulting in significant suppression of cell proliferation at 72 h. When treated with the designated concentration (5 μM for HCT116 and HCT116/5FUR, and 30 μM for DLD‐1 and DLD‐1/5FUR) of FTD, cell viability at 72 h, normalized to the corresponding control (set as 1), was numerically higher in the 5‐FU–resistant sublines than in the corresponding parental cell lines. However, in the resistant sublines, the addition of AZD6738 produced a level of growth inhibition that appeared comparable to that observed with FTD monotherapy in the corresponding parental cell lines (Figure 2B). As Figure 2A and Figure 2B correspond to different experimental designs (dose–response analysis vs. single‐concentration time‐course analysis), these datasets provide complementary rather than directly comparable measures of the combination effect.

### 3.5 DNA Damage, Apoptosis, and Chk1 Phosphorylation

**FIGURE 3:**
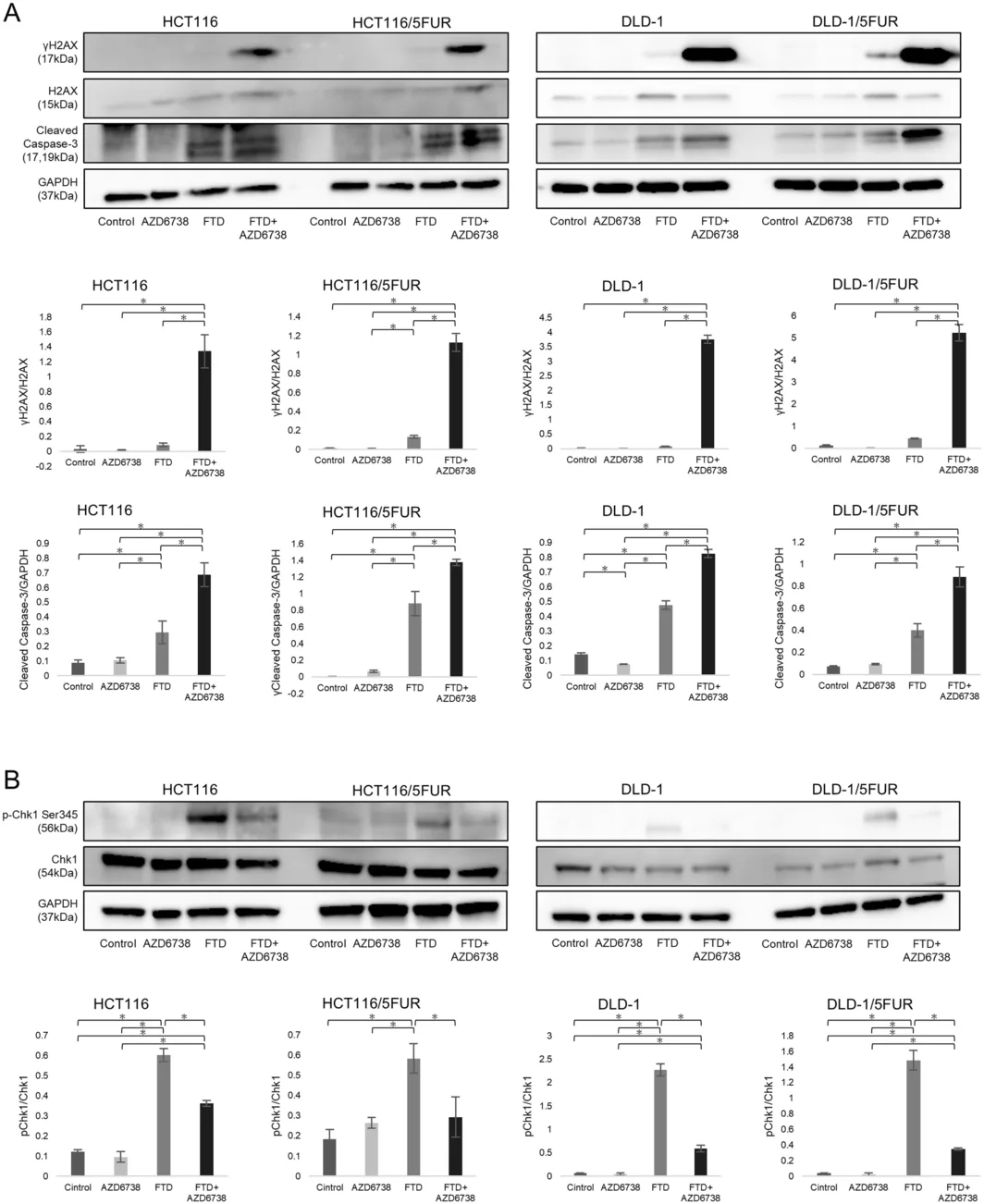
Western blots of FTD + AZD6738. HCT116, HCT116/5FUR, DLD‐1, and DLD‐1/5FUR cells were treated as indicated; γH2AX and cleaved caspase‐3 were assessed at 48 h and p‐Chk1 (Ser345) at 24 h. (A) The AZD6738 + FTD combination markedly increased γH2AX and strongly elevated cleaved caspase‐3. (B) AZD6738 suppressed the FTD‐induced increase in p‐Chk1. FTD, trifluridine; FUR, fluorouracil resistant.

DNA damage and apoptosis were assessed 48 h posttreatment by Western blotting for γH2AX and cleaved caspase‐3, respectively, while Chk1 phosphorylation at Ser345 (p‐Chk1 Ser345) was evaluated 24 h posttreatment. FTD alone produced little γH2AX induction, whereas cotreatment with AZD6738 elicited a marked increase. Cleaved caspase‐3 levels increased more strongly with the combination than with FTD alone, indicating apoptosis (Figure 3A). Moreover, the FTD‐induced increase in p‐Chk1 was suppressed following treatment with AZD6738 (Figure 3B).

### 3.6 Cell‐Cycle Analysis

**FIGURE 4:**
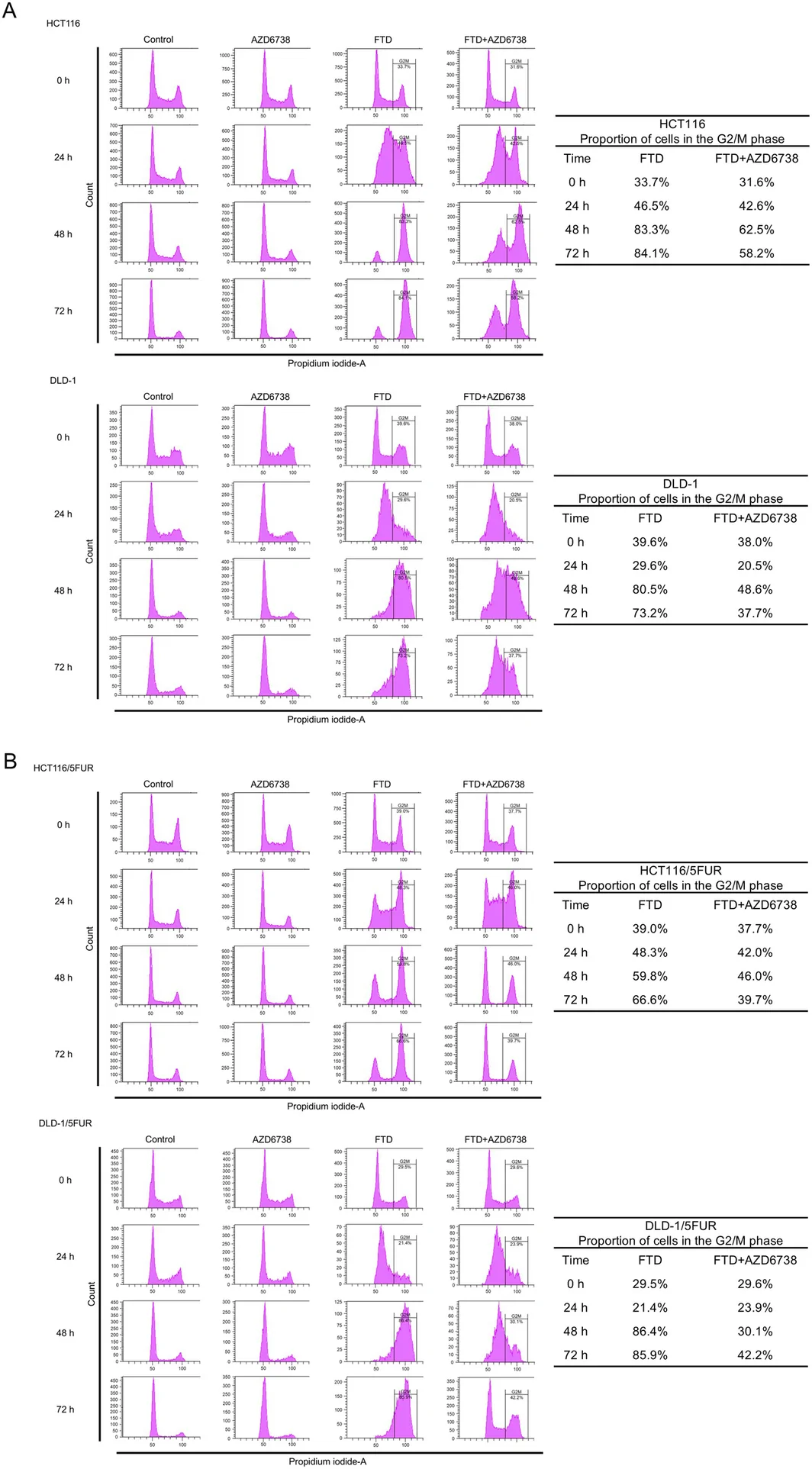
The G2/M checkpoint inhibitor AZD6738 abrogates FTD‐induced G2/M arrest in parental and 5‐FU–resistant cell lines. Both parental (A) and 5‐FU–resistant (B) colorectal cancer cell lines were treated under the indicated conditions, and cell‐cycle distributions were analyzed by flow cytometry at 0, 24, 48, and 72 h. AZD6738 alone produced no appreciable change in phase distribution. FTD monotherapy induced a marked accumulation of cells in G2/M (G2/M arrest), which was relieved by cotreatment with AZD6738. FTD, trifluridine; FUR, fluorouracil resistant.

**FIGURE 5:**
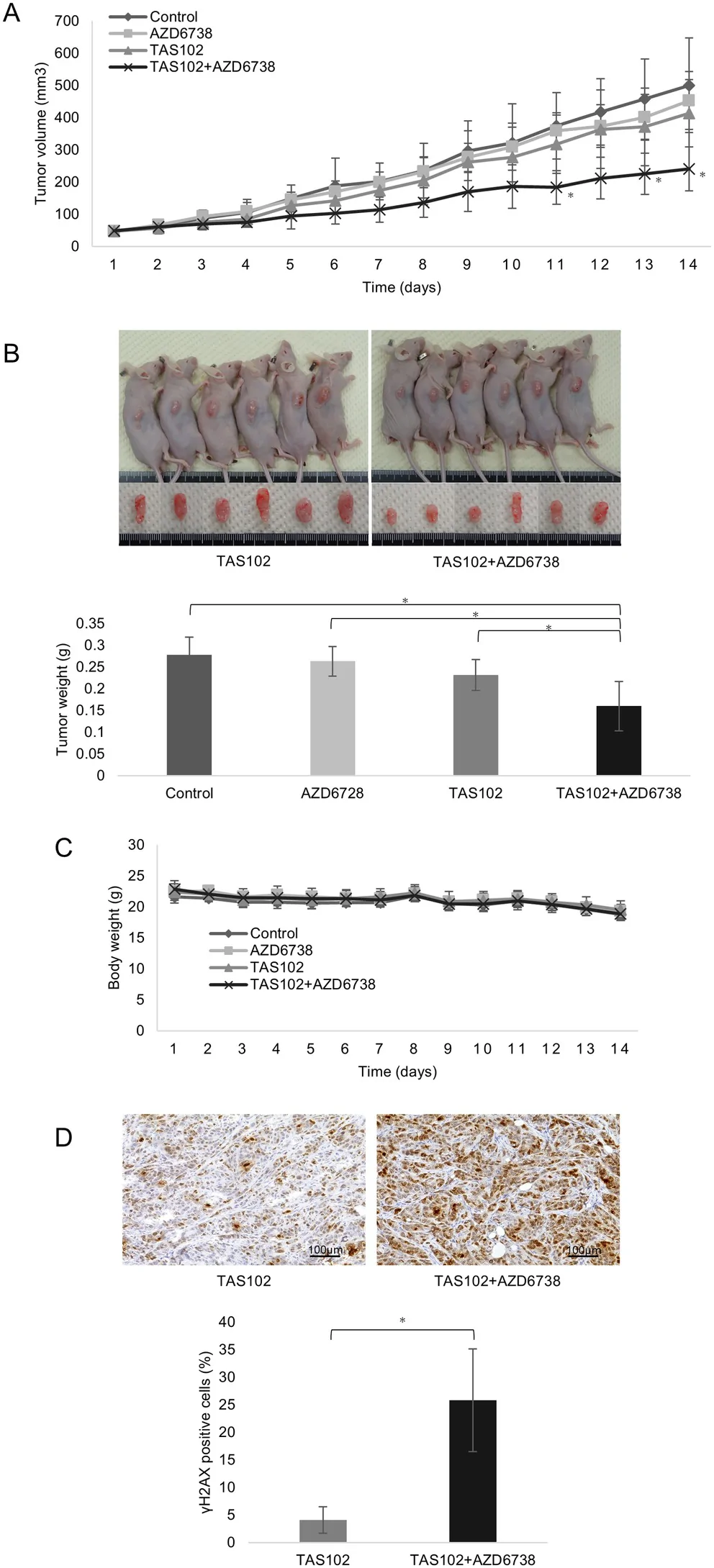
Animal study in the DLD‐1/5FUR mouse xenograft model. (A) TAS‐102 monotherapy did not significantly reduce tumor volume versus control or AZD6738 alone, whereas TAS‐102 + AZD6738 significantly suppressed tumor growth and resulted in significantly lower tumor volumes than TAS‐102 monotherapy (*p* = 0.007). (B) Similarly, at necropsy, tumor weight was significantly lower in the combination group than in the control (*p* = 5.7 × 10^−4^), AZD6738 monotherapy (*p* = 0.002), and FTD monotherapy (*p* = 0.039) groups. (C) Body weight did not differ among groups. (D) Immunohistochemistry for γH2AX showed a higher proportion of positive cells with the combination than with TAS‐102 alone (25.8% vs. 4.1%, *p* = 2.5 × 10^−4^). Data indicate mean ± SD. Asterisks indicate *p* < 0.05. TAS‐102, trifluridine + tipiracil.

Flow cytometry revealed G2/M arrest under FTD monotherapy whereas the G2/M fraction decreased with the FTD + AZD6738 combination in both parental (Figure 4A) and 5‐FU–resistant cells (Figure 4B). The cell accumulation in the G1 phase observed upon FTD treatment in p53‐wild‐type HCT116 was not detected in p53‐mutant DLD‐1.

### 3.7 In Vivo Antitumor Effect and Safety

Figure 5 shows the results from the DLD‐1/5FUR mouse xenograft model. Figure 5A shows the time course of tumor volumes. Neither AZD6738 nor TAS‐102 monotherapy produced significant differences compared with the control group. In contrast, TAS‐102 + AZD6738 significantly suppressed tumor growth over the 2‐week treatment period compared with the other groups. Similarly, at necropsy, only the combination group exhibited a significant reduction in tumor weight relative to the other groups (Figure 5B). In contrast, there were no significant differences in body weight among groups (Figure 5C). Organ weights and blood cell counts showed no increase in adverse events in the TAS‐102 + AZD6738 group versus TAS‐102 alone, indicating preserved safety (Figure S2). Immunohistochemistry staining for γH2A.X in tumors showed a significantly higher proportion of positive cells in the combination group (25.8%) than in the FTD monotherapy group (4.1%) (Figure 5D).

## 4 Discussion

Our previous studies demonstrated that pharmacological ATR inhibition with AZD6738 enhanced the antitumor effects of both 5‐FU and FTD in treatment‐naïve CRC cell lines [23, 29]. In contrast, the present study specifically focused on CRC cells with acquired resistance to 5‐FU, which represents a clinically relevant challenge during fluoropyrimidine‐based chemotherapy. Using independently established 5‐FU–resistant cell lines, we demonstrate that AZD6738 enhances the antitumor activity of FTD following acquisition of 5‐FU resistance. Thus, rather than reproducing our previous findings, the current study extends them by providing proof‐of‐concept evidence that pharmacological ATR inhibition may remain effective in a fluoropyrimidine‐resistant setting. All experiments presented in this study were performed independently of our previous reports and represent a distinct dataset. These findings extend the potential applicability of AZD6738 from treatment‐naïve CRC models to acquired 5‐FU–resistant models, although additional mechanistic studies are required.

In CRC, there is a limited number of reports on combinations of existing agents with DNA‐damage repair inhibitors. Most of these studies have focused on PARP inhibitors [38, 39]; other than our series, no prior studies have examined combinations with ATR inhibitors. In addition, to our knowledge, this is the first evaluation conducted in resistant cell lines.

5‐FU has two main functions: inhibiting thymidylate synthase (TS) and being incorporated into DNA and RNA, thereby causing DNA damage [40]. Similarly, FTD also inhibits DNA synthesis via TS and causes DNA damage through direct incorporation; however, compared with other agents, it has a particularly high incorporation rate. FTD is incorporated into DNA during S phase in place of thymidine, leading to DNA dysfunction. This damage is known to be processed via a pathway involving phosphorylation of Chk1 at Ser345 at the G2/M checkpoint [25, 41, 42].

TS overexpression has been reported to play a major role in the acquisition of 5‐FU resistance, thereby diminishing the efficacy of drugs dependent on TS inhibition [40, 43, 44, 45, 46]. Emura et al. reported that, unlike 5‐FU, 5‐fluoro‐2′‐deoxyuridine, and S‐1, FTD retained substantial cytotoxicity in 5‐FU–resistant CRC cell lines, suggesting that its antitumor activity depends largely on DNA incorporation rather than TS inhibition [34]. Accordingly, we hypothesized that combining FTD with the ATR inhibitor AZD6738 would enhance the antitumor activity of FTD even in 5‐FU–resistant cells. Although using a Chk1 inhibitor instead of an ATR inhibitor, Akasaka et al. reported that the effects of the combination of 5‐FU and a Chk1 inhibitor in CRC cells were lost in 5‐FU–resistant sublines. Thus, under acquired 5‐FU resistance, combining 5‐FU with inhibitors of DNA damage repair may not be effective [47]. The present findings suggest that pharmacological ATR inhibition with AZD6738 enhances the antitumor activity of FTD even in 5‐FU–resistant CRC cells. These observations are consistent with the reported differences in the pharmacological properties of 5‐FU and FTD. In highly 5‐FU–resistant sublines, the effect of FTD alone was modestly attenuated, possibly reflecting reduced contribution of TS inhibition after acquisition of 5‐FU resistance. Notably, the addition of AZD6738 partially restored the antitumor activity of FTD. These findings support further investigation of this combination in treatment‐refractory CRC models.

The apparent differences between Figure 2A,B do not represent inconsistent biological findings but rather reflect the different aspects of drug response evaluated by each analytical approach. IC50 values derived from dose–response curves indicate overall drug sensitivity across a range of concentrations, whereas single‐concentration viability assays assess cellular responses under a predefined experimental condition. In addition, combination index analysis quantifies the pharmacological interaction between two agents and should not be interpreted as equivalent to the magnitude of IC50 shift. Accordingly, these analyses provide complementary information and are not intended for direct quantitative comparison.

Hematologic toxicity has been a concern in previous combination studies with PARP inhibitors, paclitaxel, and gemcitabine; however, it can be manageable with dose reduction or supportive care [18, 48, 49]. In our mouse model, TAS‐102 alone induced hematologic toxicity but the addition of AZD6738 did not exacerbate this toxicity, and no body weight loss or organ damage was observed. Despite the short‐term evaluation, these findings suggest acceptable tolerability in this preclinical model.

The present study has several limitations. First, mechanistic conclusions are based solely on pharmacological ATR inhibition with AZD6738, as no genetic validation of ATR inhibition was performed. Second, normal colon epithelial cells were not evaluated; therefore, the selectivity and therapeutic window of this combination remain uncertain. Future studies should address this limitation by evaluating the effects of the combination in nonmalignant colon epithelial models. Third, only a single biologically selected concentration (0.5 μM) of AZD6738 was examined. Therefore, the conclusions of the present study are limited to this concentration, and broader dose–response studies will be required to determine the optimal biological dose. Finally, additional molecular determinants of ATR inhibitor sensitivity were not investigated. ATR inhibitors can effectively induce synthetic lethality in ATM‐deficient or p53‐deficient tumors, with reportedly limited effects in p53 wild‐type settings [50, 51]. However, some studies have demonstrated efficacy in cell lines with functional ATM [35]. In this study, synergistic effects were observed in both p53 wild‐type (HCT116/HCT116‐5FUR) and p53‐mutant (DLD‐1/DLD‐1/5FUR) CRC models. Whether p53 contributed to the responses observed in the present study remains speculative because only two cell lines with multiple molecular differences were examined and no experiments were performed to directly evaluate the role of p53. Therefore, no conclusion regarding the contribution of p53 can be drawn, and further studies are warranted to identify the molecular determinants of ATR inhibitor sensitivity.

From a clinical perspective, FTD is generally administered as part of TAS‐102 after failure of fluoropyrimidine‐based first‐ and second‐line chemotherapy. Consequently, many patients receiving TAS‐102 have already been exposed to prolonged 5‐FU treatment and may harbor acquired resistance mechanisms. The present findings provide a rationale for further investigation of pharmacological ATR inhibition in this clinically relevant setting.

In conclusion, pharmacological ATR inhibition with AZD6738 enhanced the antitumor activity of FTD in preclinical models of acquired 5‐FU resistance. These findings provide proof‐of‐concept supporting further mechanistic investigation and future translational studies.

## Author Contributions

**Shuhei Uehara:** data curation, formal analysis, validation, visualization, project administration, writing – original draft, writing – review and editing, investigation, conceptualization, methodology, resources. **Takuya Suzuki:** conceptualization, data curation, formal analysis, investigation, methodology, project administration, writing – review and editing, funding acquisition, validation. **Junki Kato:** writing – review and editing. **Hiroyuki Asai:** writing – review and editing. **Akira Kato:** writing – review and editing, investigation, data curation. **Shinnosuke Harata:** data curation, investigation, validation, writing – review and editing, conceptualization, methodology, project administration. **Hajime Ushigome:** writing – review and editing. **Yushi Yamakawa:** writing – review and editing, supervision, project administration. **Takahisa Hirokawa:** writing – review and editing, supervision, project administration. **Hiroki Takahashi:** writing – review and editing, supervision, project administration. **Yoichi Matsuo:** writing – review and editing, supervision, methodology, conceptualization, validation, project administration. **Shuji Takiguchi:** writing – review and editing, supervision.

## Funding

The present study was supported by Grants‐in‐Aid for Scientific Research (Japan Society for the Promotion of Science; 19K18158).

## Ethics Statement

In vivo mouse experiments were approved by the Animal Care and Use Committee of the Nagoya City University Graduate School of Medical Sciences (approval no. 23‐049; Nagoya, Japan).

## Consent

The authors have nothing to report.

## Conflicts of Interest

The authors declare no conflicts of interest.

## Supporting information


**Figure S1:** WST‐1 assessment of FTD sensitivity in parental versus 5‐FU–resistant CRC cell lines.

**Figure S2:** Organ weights and hematology.

## Data Availability

The data that support the findings of this study are available from the corresponding author upon reasonable request.

## References

[1] E. Morgan , M. Arnold , A. Gini , et al., “Global Burden of Colorectal Cancer in 2020 and 2040: Incidence and Mortality Estimates From GLOBOCAN,” Gut 72, no. 2 (2023): 338–344, 10.1136/gutjnl-2022-327736. 36604116

[2] C. P. Wild , E. Weiderpass , and B. W. Stewart , IARC World Cancer Report: Cancer Research for Cancer Prevention (International Agency for Research on Cancer, 2020). 39432694

[3] K. Matsumoto , Y. Hatakeyama , K. Seto , et al., “Cost of Illness for Colorectal Cancer in Japan—A Time Trend and Future Projections (1996–2035) Based on Governmental Statistics,” BMC Health Services Research 23, no. 1 (2023): 888, 10.1186/s12913-023-09831-8. 37608367 PMC10463985

[4] Y. Hashiguchi , K. Muro , Y. Saito , et al., “Japanese Society for Cancer of the Colon and Rectum (JSCCR) Guidelines 2019 for the Treatment of Colorectal Cancer,” International Journal of Clinical Oncology 25, no. 1 (2020): 1–42, 10.1093/annonc/mdx572. 31203527 PMC6946738

[5] T. André , C. Boni , L. Mounedji‐Boudiaf , et al., “Oxaliplatin, Fluorouracil, and Leucovorin as Adjuvant Treatment for Colon Cancer,” New England Journal of Medicine 350, no. 23 (2004): 2343–2351, 10.1056/nejmoa032709. 15175436

[6] E. Van Cutsem , C. H. Köhne , E. Hitre , et al., “Cetuximab and Chemotherapy as Initial Treatment for Metastatic Colorectal Cancer,” New England Journal of Medicine 360, no. 14 (2009): 1408–1417, 10.1056/nejmoa0805019. 19339720

[7] H. Hurwitz , L. Fehrenbacher , W. Novotny , et al., “Bevacizumab Plus Irinotecan, Fluorouracil, and Leucovorin for Metastatic Colorectal Cancer,” New England Journal of Medicine 350, no. 23 (2004): 2335–2342, 10.1056/NEJMoa032691. 15175435

[8] A. P. Venook , D. Niedzwiecki , H. J. Lenz , et al., “Effect of First‐Line Chemotherapy Combined With Cetuximab or Bevacizumab on Overall Survival in Patients With KRAS Wild‐Type Advanced or Metastatic Colorectal Cancer,” Journal of the American Medical Association 317, no. 23 (2017): 2392–2401, 10.1001/jama.2017.7105. 28632865 PMC5545896

[9] A. R. Cuddihy and M. J. O'Connell , “Cell‐Cycle Responses to DNA Damage in G2,” International Review of Cytology 222 (2003): 99–140, 10.1016/s0074-7696(02)22013-6. 12503848

[10] T. Hirokawa , B. Shiotani , M. Shimada , et al., “CBP‐93872 Inhibits NBS1‐Mediated ATR Activation, Abrogating Maintenance of the DNA Double‐Strand Break‐Specific G2 Checkpoint,” Cancer Research 74, no. 14 (2014): 3880–3889, 10.1158/0008-5472.can-13-3604. 24876101

[11] Z. Qiu , N. L. Oleinick , and J. Zhang , “ATR/CHK1 Inhibitors and Cancer Therapy,” Radiotherapy and Oncology 126, no. 3 (2018): 450–464, 10.1016/j.radonc.2017.09.043. 29054375 PMC5856582

[12] Y. J. Shim , “Therapeutic Targeting of DNA Damage Response Pathways in TP53‐ and ATM‐Mutated Tumors,” Brain Tumor Research and Treatment 13, no. 3 (2025): 73–80, 10.14791/btrt.2025.0017. 40759474 PMC12329235

[13] Z. Wilson , R. Odedra , Y. Wallez , et al., “ATR Inhibitor AZD6738 (Ceralasertib) Exerts Antitumor Activity as a Monotherapy and in Combination With Chemotherapy and the PARP Inhibitor Olaparib,” Cancer Research 82, no. 6 (2022): 1140–1152, 10.1158/0008-5472.can-21-2997. 35078817 PMC9359726

[14] A. Min , S. A. Im , H. Jang , et al., “AZD6738, A Novel Oral Inhibitor of ATR, Induces Synthetic Lethality With ATM Deficiency in Gastric Cancer Cells,” Molecular Cancer Therapeutics 16, no. 4 (2017): 566–577, 10.1158/1535-7163.mct-16-0378. 28138034

[15] L. Mei , J. Zhang , K. He , and J. Zhang , “Ataxia Telangiectasia and Rad3‐Related Inhibitors and Cancer Therapy: Where We Stand,” Journal of Hematology & Oncology 12, no. 1 (2019): 43, 10.1186/s13045-019-0733-6. 31018854 PMC6482552

[16] Y. Wallez , C. R. Dunlop , T. I. Johnson , et al., “The ATR Inhibitor AZD6738 Synergizes With Gemcitabine In Vitro and In Vivo to Induce Pancreatic Ductal Adenocarcinoma Regression,” Molecular Cancer Therapeutics 17, no. 8 (2018): 1670–1682, 10.1158/1535-7163.mct-18-0010. 29891488 PMC6076438

[17] A. R. Nam , M. H. Jin , J. E. Park , J. H. Bang , D. Y. Oh , and Y. J. Bang , “Therapeutic Targeting of the DNA Damage Response Using an ATR Inhibitor in Biliary Tract Cancer,” Cancer Research and Treatment 51, no. 3 (2019): 1167–1179, 10.4143/crt.2018.526. 30514066 PMC6639230

[18] M. Kwon , G. Kim , R. Kim , et al., “Phase II Study of Ceralasertib (AZD6738) in Combination With Durvalumab in Patients With Advanced Gastric Cancer,” Journal for Immunotherapy of Cancer 10, no. 7 (2022): e005041, 10.1136/jitc-2022-005041. 35790315 PMC9258491

[19] B. Besse , E. Pons‐Tostivint , K. Park , et al., “Biomarker‐Directed Targeted Therapy Plus Durvalumab in Advanced Non‐Small‐Cell Lung Cancer: A Phase 2 Umbrella Trial,” Nature Medicine 30, no. 3 (2024): 716–729, 10.1038/s41591-024-02808-y. PMC10957481 38351187

[20] A. Ring , L. S. Kilburn , A. Pearson , et al., “Olaparib and Ceralasertib (AZD6738) in Patients With Triple‐Negative Advanced Breast Cancer: Results From Cohort E of the plasmaMATCH Trial (CRUK/15/010),” Clinical Cancer Research 29, no. 23 (2023): 4751–4759, 10.1158/1078-0432.ccr-23-1696. 37773077 PMC10690092

[21] R. Kim , M. Kwon , M. An , et al., “Phase II Study of Ceralasertib (AZD6738) in Combination With Durvalumab in Patients With Advanced/Metastatic Melanoma Who Have Failed Prior Anti‐PD‐1 Therapy,” Annals of Oncology 33, no. 2 (2022): 193–203, 10.1016/j.annonc.2021.10.009. 34710570

[22] M. T. Dillon , J. Guevara , K. Mohammed , et al., “Durable Responses to ATR Inhibition With Ceralasertib in Tumors With Genomic Defects and High Inflammation,” Journal of Clinical Investigation 134, no. 2 (2024): e175369, 10.1172/jci175369. 37934611 PMC10786692

[23] T. Suzuki , T. Hirokawa , A. Maeda , et al., “ATR Inhibitor AZD6738 Increases the Sensitivity of Colorectal Cancer Cells to 5‐Fluorouracil by Inhibiting Repair of DNA Damage,” Oncology Reports 47, no. 4 (2022): 78, 10.3892/or.2022.8289. 35191521 PMC8892626

[24] T. Emura , F. Nakagawa , A. Fujioka , et al., “An Optimal Dosing Schedule for a Novel Combination Antimetabolite, TAS‐102, Based on Its Intracellular Metabolism and Its Incorporation Into DNA,” International Journal of Molecular Medicine 13, no. 2 (2004): 249–255, 10.3892/ijmm.13.2.249. 14719131

[25] N. Suzuki , T. Emura , and M. Fukushima , “Mode of Action of Trifluorothymidine (TFT) Against DNA Replication and Repair Enzymes,” International Journal of Oncology 39, no. 1 (2011): 263–270, 10.3892/ijo.2011.1003. 21491084

[26] R. J. Mayer , E. Van Cutsem , A. Falcone , et al., “Randomized Trial of TAS‐102 for Refractory Metastatic Colorectal Cancer,” New England Journal of Medicine 372, no. 20 (2015): 1909–1919, 10.1056/nejmoa1414325. 25970050

[27] G. W. Prager , J. Taieb , M. Fakih , et al., “Trifluridine–Tipiracil and Bevacizumab in Refractory Metastatic Colorectal Cancer,” New England Journal of Medicine 388, no. 18 (2023): 1657–1667, 10.1056/nejmoa2214963. 37133585

[28] Japanese Society for Cancer of the Colon and Rectum (JSCCR) , JSCCR Guidelines 2024 for the Treatment of Colorectal Cancer (Kanehara & Co., Ltd, 2024). 10.1007/s10147-025-02899-8 PMC12644182 41186794

[29] S. Harata , T. Suzuki , H. Takahashi , et al., “AZD6738 Promotes the Tumor Suppressive Effects of Trifluridine in Colorectal Cancer Cells,” Oncology Reports 49, no. 3 (2023): 52, 10.3892/or.2023.8489. 36734271 PMC9926513

[30] S. Blondy , V. David , M. Verdier , M. Mathonnet , A. Perraud , and N. Christou , “5‐Fluorouracil Resistance Mechanisms in Colorectal Cancer: From Classical Pathways to Promising Processes,” Cancer Science 111, no. 9 (2020): 3142–3154, 10.1111/cas.14532. 32536012 PMC7469786

[31] T. Suetsugu , R. Mori , M. Futamura , et al., “Mechanism of Acquired 5FU Resistance and Strategy for Overcoming 5FU Resistance Focusing on 5FU Metabolism in Colon Cancer Cell Lines,” Oncology Reports 45, no. 4 (2021): 27, 10.3892/or.2021.7978. 33649846 PMC7905524

[32] Y. Murakami , H. Kazuno , T. Emura , H. Tsujimoto , N. Suzuki , and M. Fukushima , “Different Mechanisms of Acquired Resistance to Fluorinated Pyrimidines in Human Colorectal Cancer Cells,” International Journal of Oncology 17, no. 2 (2000): 277–283, 10.3892/ijo.17.2.277. 10891536

[33] M. Fukushima , N. Suzuki , T. Emura , et al., “Structure and Activity of Specific Inhibitors of Thymidine Phosphorylase to Potentiate the Function of Antitumor 2′‐Deoxyribonucleosides,” Biochemical Pharmacology 59, no. 10 (2000): 1227–1236, 10.1016/s0006-2952(00)00253-7. 10736423

[34] T. Emura , N. Suzuki , M. Yamaguchi , H. Ohshimo , and M. Fukushima , “A Novel Combination Antimetabolite, TAS‐102, Exhibits Antitumor Activity in FU‐Resistant Human Cancer Cells Through a Mechanism Involving FTD Incorporation in DNA,” International Journal of Oncology 25, no. 3 (2004): 571–578, 10.3892/ijo.25.3.571. 15289858

[35] F. P. Vendetti , A. Lau , S. Schamus , T. P. Conrads , M. J. O'Connor , and C. J. Bakkenist , “The Orally Active and Bioavailable ATR Kinase Inhibitor AZD6738 Potentiates the Anti‐Tumor Effects of Cisplatin to Resolve ATM‐Deficient Non‐Small Cell Lung Cancer In Vivo,” Oncotarget 6, no. 42 (2015): 44289–44305, 10.18632/oncotarget.6247. 26517239 PMC4792557

[36] S. Checkley , L. MacCallum , J. Yates , et al., “Bridging the Gap Between In Vitro and In Vivo: Dose and Schedule Predictions for the ATR Inhibitor AZD6738,” Scientific Reports 5, no. 1 (2015): 13545, 10.1038/srep13545. 26310312 PMC4550834

[37] T. C. Chou and P. Talalay , “Quantitative Analysis of Dose–Effect Relationships: The Combined Effects of Multiple Drugs or Enzyme Inhibitors,” Advances in Enzyme Regulation 22 (1984): 27–55, 10.1016/0065-2571(84)90007-4. 6382953

[38] H. de Castro E Gloria , L. Jesuíno Nogueira , P. Bencke Grudzinski , et al., “Olaparib‐Mediated Enhancement of 5‐Fluorouracil Cytotoxicity in Mismatch Repair Deficient Colorectal Cancer Cells,” BMC Cancer 21, no. 1 (2021): 448, 10.1186/s12885-021-08188-7. 33888065 PMC8063290

[39] A. Jarrar , F. Lotti , J. DeVecchio , et al., “Poly(ADP‐Ribose) Polymerase Inhibition Sensitizes Colorectal Cancer‐Initiating Cells to Chemotherapy,” Stem Cells 37, no. 1 (2019): 42–53, 10.1002/stem.2929. 30353615

[40] D. B. Longley , D. P. Harkin , and P. G. Johnston , “5‐Fluorouracil: Mechanisms of Action and Clinical Strategies,” Nature Reviews. Cancer 3, no. 5 (2003): 330–338, 10.1038/nrc1074. 12724731

[41] N. Tanaka , K. Sakamoto , H. Okabe , et al., “Repeated Oral Dosing of TAS‐102 Confers High Trifluridine Incorporation Into DNA and Sustained Antitumor Activity in Mouse Models,” Oncology Reports 32, no. 6 (2014): 2319–2326, 10.3892/or.2014.3487. 25230742 PMC4240496

[42] K. Matsuoka , M. Iimori , S. Niimi , et al., “Trifluridine Induces p53‐Dependent Sustained G2 Phase Arrest With Its Massive Misincorporation Into DNA and Few DNA Strand Breaks,” Molecular Cancer Therapeutics 14, no. 4 (2015): 1004–1013, 10.1158/1535-7163.mct-14-0236. 25700705

[43] S. Copur , K. Aiba , J. C. Drake , C. J. Allegra , and E. Chu , “Thymidylate Synthase Gene Amplification in Human Colon Cancer Cell Lines Resistant to 5‐Fluorouracil,” Biochemical Pharmacology 49, no. 10 (1995): 1419–1426, 10.1016/0006-2952(95)00067-a. 7763285

[44] J. Y. Ahn , J. S. Lee , H. Y. Min , and H. Y. Lee , “Acquired Resistance to 5‐Fluorouracil via HSP90/Src‐Mediated Increase in Thymidylate Synthase Expression in Colon Cancer,” Oncotarget 6, no. 32 (2015): 32622–32633, 10.18632/oncotarget.5327. 26416450 PMC4741717

[45] G. J. Peters , H. H. Backus , S. Freemantle , et al., “Induction of Thymidylate Synthase as a 5‐Fluorouracil Resistance Mechanism,” Biochimica et Biophysica Acta 1587, no. 2–3 (2002): 194–205, 10.1016/s0925-4439(02)00082-0. 12084461

[46] Y. Saga , M. Suzuki , H. Mizukami , et al., “Overexpression of Thymidylate Synthase Mediates Desensitization for 5‐Fluorouracil of Tumor Cells,” International Journal of Cancer 106, no. 3 (2003): 324–326, 10.1002/ijc.11221. 12845668

[47] T. Akasaka , M. Tsujii , J. Kondo , et al., “5‐FU Resistance Abrogates the Amplified Cytotoxic Effects Induced by Inhibiting Checkpoint Kinase 1 in p53‐Mutated Colon Cancer Cells,” International Journal of Oncology 46, no. 1 (2015): 63–70, 10.3892/ijo.2014.2693. 25310623

[48] S. T. Kim , S. A. Smith , P. Mortimer , et al., “Phase I Study of Ceralasertib (AZD6738), A Novel DNA Damage Repair Agent, in Combination With Weekly Paclitaxel in Refractory Cancer,” Clinical Cancer Research 27, no. 17 (2021): 4700–4709, 10.1158/1078-0432.ccr-21-0251. 33975862 PMC8974415

[49] T. A. Yap , M. G. Krebs , S. Postel‐Vinay , et al., “Ceralasertib (AZD6738), an Oral ATR Kinase Inhibitor, in Combination With Carboplatin in Patients With Advanced Solid Tumors: A Phase I Study,” Clinical Cancer Research 27, no. 19 (2021): 5213–5224, 10.1158/1078-0432.ccr-21-1032. 34301752 PMC9401487

[50] E. Durinikova , N. M. Reilly , K. Buzo , et al., “Targeting the DNA Damage Response Pathways and Replication Stress in Colorectal Cancer,” Clinical Cancer Research 28, no. 17 (2022): 3874–3889, 10.1158/1078-0432.ccr-22-0875. 35881546 PMC9433963

[51] N. J. Curtin , “Targeting the DNA Damage Response for Cancer Therapy,” Biochemical Society Transactions 51, no. 1 (2023): 207–221, 10.1042/bst20220681. 36606678 PMC9988002

